# Direct susceptibility testing for multi drug resistant tuberculosis: A meta-analysis

**DOI:** 10.1186/1471-2334-9-67

**Published:** 2009-05-20

**Authors:** Freddie Bwanga, Sven Hoffner, Melles Haile, Moses L Joloba

**Affiliations:** 1 Department of Medical Microbiology Makerere University College of Health Sciences Kampala, Uganda; 2 Department of Bacteriology, Swedish Institute for Infectious Disease Control, Solna, Sweden; 3 Department of Microbiology, Tumour and Cell Biology (MTC), Karolinska Institute, Stockholm, Sweden

## Abstract

**Background:**

One of the challenges facing the tuberculosis (TB) control programmes in resource-limited settings is lack of rapid techniques for detection of drug resistant TB, particularly multi drug resistant tuberculosis (MDR TB). Results obtained with the conventional indirect susceptibility testing methods come too late to influence a timely decision on patient management. More rapid tests directly applied on sputum samples are needed. This study compared the sensitivity, specificity and time to results of four direct drug susceptibility testing tests with the conventional indirect testing for detection of resistance to rifampicin and isoniazid in *M. tuberculosis*. The four direct tests included two in-house phenotypic assays – Nitrate Reductase Assay (NRA) and Microscopic Observation Drug Susceptibility (MODS), and two commercially available tests – Genotype^® ^MTBDR and Genotype^® ^MTBDR*plus *(Hain Life Sciences, Nehren, Germany).

**Methods:**

A literature review and meta-analysis of study reports was performed. The Meta-Disc software was used to analyse the reports and tests for sensitivity, specificity, and area under the summary receiver operating characteristic (sROC) curves. Heterogeneity in accuracy estimates was tested with the Spearman correlation coefficient and Chi-square.

**Results:**

Eighteen direct DST reports were analysed: NRA – 4, MODS- 6, Genotype MTBDR^® ^– 3 and Genotype^® ^MTBDR*plus *– 5. The pooled sensitivity and specificity for detection of resistance to rifampicin were 99% and 100% with NRA, 96% and 96% with MODS, 99% and 98% with Genotype^® ^MTBDR, and 99% and 99% with the new Genotype^® ^MTBDR*plus*, respectively. For isoniazid it was 94% and 100% for NRA, 92% and 96% for MODS, 71% and 100% for Genotype^® ^MTBDR, and 96% and 100% with the Genotype^® ^MTBDR*plus*, respectively. The area under the summary receiver operating characteristic (sROC) curves was in ranges of 0.98 to 1.00 for all the four tests. Molecular tests were completed in 1 – 2 days and also the phenotypic assays were much more rapid than conventional testing.

**Conclusion:**

Direct testing of rifampicin and isoniazid resistance in *M. tuberculosis *was found to be highly sensitive and specific, and allows prompt detection of MDR TB.

## Background

Tuberculosis (TB) continues to be a leading cause of morbidity and mortality in developing countries [1]. Global efforts for TB control are being challenged by the steady increase in drug-resistant TB, particularly multidrug resistant tuberculosis (MDR TB), defined as resistance to at least rifampicin (RIF) and isoniazid (INH). The World Health Organization (WHO) estimates that 500,000 new cases of MDR TB occur globally every year and MDR TB has been reported in 2.9% and 15.3% among the new and previously treated cases, respectively [2].

MDR TB requires 18–24 months of treatment with expensive second line drugs some of which are injectable agents. The cure rate is much lower than for drug susceptible TB, only around 60% [3]. Therefore, it is crucial that MDR TB should be detected as soon as possible, and measures implemented to effectively control its further spread.

Conventional methods for detection of MDR TB involve primary culture of specimens and isolation of *Mycobacterium tuberculosis *(MTB), followed by drug susceptibility testing (DST). This process, referred to as indirect susceptibility testing has a long turn around time (TAT) of around 2 months. The TAT is longest in the TB high burden low-income countries where primary isolation and indirect DST are almost exclusively performed on solid medium. Use of liquid systems such as the BACTEC MGIT 960 system (Becton Dickinson, Sparks, Maryland, USA) has improved TAT to about 25–45 days, but liquid culture systems are in most cases not available where the need is greatest [4].

Even though liquid-based indirect susceptibility tests have improved the TAT, they are still not rapid enough to allow timely decisions on patient management in case of MDR TB. More rapid TB susceptibility tests are needed, particularly in TB high burden countries. Recently, the focus has shifted to rapid direct tests in which decontaminated respiratory samples are directly inoculated in drug-free and drug-containing medium or amplified for detection of MDR TB. Some of the direct tests being studied with prospects for applicability in developing countries include the Nitrate Reductase Assay (NRA); Microscopic Observation Drug Susceptibility (MODS) assay, and more recently molecular assays such as the Genotype^® ^MTBDR (Hain Life sciences, Nehren, Germany), and its newer version – the Genotype^® ^MTBDR*plus*.

The **NRA **test, initially introduced as an indirect assay is performed on solid medium as for the proportion method, though liquid-based assays have recently been studied [5-9]. The medium is supplemented with potassium or sodium nitrate at a concentration of 1000 mg/L to act as a growth indicator. Live *M. tuberculosis *organisms possess the nitro-reductase enzyme and will reduce nitrate to nitrite, which is then detected as a pink-purple colour when a detection reagent (Griess reagent) is added to the tube [5]. A colour change in a drug-containing tube indicates resistance. The **MODS **assay is a low-technology liquid culture system performed in OADC-supplemented 7H9 broth on an ordinary tissue culture plate [10]. A cock-tail of antibiotics – polymyxin B, amphotericin B, Nalidixic acid, trimethoprim and azlocillin (PANTA) is added to prevent growth of contaminating bacteria and fungi. Incorporation of isoniazid and rifampicin in the wells followed by inoculation of processed samples in the drug-free and drug containing wells allows direct detection of MDR TB. When *M. tuberculosis *grows in the broth, characteristic cord-like structures can be seen under an inverted microscope, permitting early detection of resistance [10-16]. The MODS assay has been studied on both smear positive and smear negative sputum samples with good results [11], which is not the case with any other tests. The **GenoType**^®^**MTBDR **assay is a molecular test that detects the common mutations in the *rpoB *and *katG *genes responsible for resistance to rifampicin and isoniazid, respectively [17]. The test involves DNA extraction, multiplex polymerase chain reaction (PCR), solid phase reverse hybridization and detection of the resistance mutations [18-20]. The Genotype^® ^MTBDR*plus *assay detects additional mutations in the *rpoB *gene and also in the *inhA *gene promoter region, giving a higher sensitivity in resistance detection [18,21-24].

Published studies have evaluated the performance of direct testing with the above mentioned tests. However, the data is spread in many different journals, which makes it difficult to fully understand the performance of direct testing, thereby delaying decisions on adoption of this approach for prompt detection of MDR TB. In this study, available data from individual study reports on direct testing with the NRA, MODS, Genotype^® ^MTBDR and Genotype^® ^MTBDR*plus *was pooled and analysed for sensitivity, specificity and time to results of direct testing against conventional indirect susceptibility testing in detection of MDR TB. The results of this meta-analysis are intended to guide TB control programmes in TB high burden countries to select for further operational study, highly sensitive and specific rapid tests to identify MDR TB.

## Methods

### Study design

A literature review and meta-analysis was conducted.

### Search strategy

Original articles published in English up to end of January 2009 were searched with PubMed and Google. Each of the four tests was searched by its name, and the name combined with the words 'tuberculosis drug resistance testing', 'rifampicin resistant tuberculosis', 'isoniazid resistant tuberculosis', 'multi drug resistant tuberculosis testing'. New links displayed beside the abstracts were followed and retrieved. Finally, the bibliographies of each article were carefully reviewed and relevant articles also retrieved. A search in other databases did not reveal any additional articles previously missed on PubMed or Google searches.

### Inclusion and exclusion criteria

Only study reports that had evaluated direct DST for detection of resistance to RIF and/or INH in *M. tuberculosis *were included. At least 3 independent direct DST reports were required to qualify a test for the pooled data analysis. Additionally, the study report must have had extractable data to fill the 4 cells of a 2 × 2 table for diagnostic tests (true resistant – TR, false resistant – FR, false susceptible – FS and true susceptible – TS). Lastly, studies were included if the reference standard test in the report was an indirect assay *i.e*. proportion method (PM) on Lowenstein-Jensen (L-J) or 7H10 agar, BACTEC 460, BACTEC MGIT 960 or a MIC (minimum inhibitory concentration) test. One genotypic study used DNA sequencing as the reference test but was also included. Indirect DST assay reports or study reports that used the test for reasons other than DST were excluded from further analysis, as were study reports without extractable data for a 2 × 2 table.

### Quality of study reports

In a meta-analysis of diagnostic accuracy studies, factors such as study design, patient selection criteria, reference standard and blinding, may be related to overly optimistic estimates of diagnostic accuracy [25]. We applied the quality of diagnostic accuracy studies tool (QUADAS tool) [26] to assess the quality of the reports included in this analysis. The QUADAS tool has 14 items that assess study design-related issues, and the internal and external validity of the results of the study [26]. Each item may be scored 'yes' if reported; 'no' if not reported; or 'unclear' if the information in the article is inadequate to make an accurate judgement.

### Data extraction

Data from study reports was extracted twice. Data items included author(s); year of publication; reference standard test; country where the study was conducted; sample size; specimen type; values of true resistance (TR), false resistance (FR), false susceptible (FS) and true susceptible (TS); and the QUADAS items. The time to results (TTR) in days from setting the test to obtaining results for 100% of the samples in each study report was recorded. The average time for each test type was then calculated.

### Data analysis

#### Accuracy estimates

Sensitivity, specificity, forest plots and summary receiver operating characteristic (sROC) curves were analysed with the Meta-Disc software, based on the fixed model effect [27]. Sensitivity was defined as the proportion of drug resistant TB strains correctly identified by the new test (true resistant rate – TRR). Specificity was defined as the proportion of susceptible isolates correctly identified by the new test (true susceptible rate – TSR).

#### Statistical testing for heterogeneity in accuracy estimates

Threshold/cut off effect as a possible cause of variations in sensitivity and specificity among the reviewed reports was tested with the Spearman correlation coefficient between the logit of sensitivity and logit of 1-specificity. Variation due to factors other than threshold/cut off effect was tested by visual inspection of the forest plots for (i) degree of deviation of sensitivity and specificity of each study from the vertical line corresponding with the pooled estimates, (ii) Chi-square *p*-values and (iii) inconsistence index.

#### Time to results

The average time to results was computed in MS office excel 2007.

## Results

Sixty-four reports were initially reviewed. Nineteen of these had studied direct DST for detection of resistance to rifampicin and/or isoniazid in *M. tuberculosis*. Eighteen of the 19 reports fulfilled the inclusion criteria for the meta-analysis. The study reports reviewed and meta-analysed or excluded, plus reasons for the exclusion are shown in table 1. The description of the 18 meta-analysed reports is given in table 2.

**Table 1:** Study reports reviewed and meta-analysed or excluded

			Excluded reports, and reason				
Test	Reviewed reports	Analysed reports	No data for 2 × 2 table	Indirect DST	Diagnostic study	Review study	*Other reasons
NRA	22	4	0	16	0	2	0
MODS	19	6	0	2	7	1	3
Genotype^® ^MTBDR	13	3	1	7	0	1	1
Genotype^® ^MTBDR*plus*	10	5	0	5	0	0	0
**Total**	**64**	**18**	**1**	**30**	**7**	**4**	**4**

*Other reasons: Three MODS studies were excluded as they were either disinfection, impact on clinical decision-making or operational issues studies. The one study under Genotype MTBDR was a hetero-resistance study.

***Abbreviations: ***DST = Drug Susceptibility Testing; MODS = Microscopic Observation Drug Susceptibility; NRA = Nitrate Reductase Assay.

**Table 2:** Description of meta-analysed reports (n = 18)

Test	Author, (Year)	Ref	Reference test	Country	Sample Size	Rifampicin				Isoniazid				Time (Days)
						TR	FR	FS	TS	TR	FR	FS	TS	
**NRA**	Affolabi D (2008)	[6]	L-J PM	Benin	144	6	1	0	137	14	1	0	129	18
	Affolabi D (2007)	[7]	L-J PM	Benin	177	7	0	1	169	**0**	**0**	**0**	**0**	28
	Solis LA (2005)	[8]	L-J PM	Peru	192	113	0	1	78	101	0	7	84	28
	Musa HR (2005)	[9]	L-J PM	Argentina	121	11	0	0	110	13	0	1	107	18
	
	**TOTAL**				**634**	**137**	**1**	**2**	**494**	**128**	**1**	**8**	**320**	

**MODS**	Ejigu GS (2008)	[10]	MGIT 960	Ethopia	58	19	0	1	38	32	2	1	23	15
	Mello FCQ (2007)	[13]	L-J PM	Honduras	180	72	18	3	87	89	19	3	69	24
	Shiferaw G (2007)	[14]	Agar PM	Ethopia	247/246	24	1	2	220	50	6	3	187	29
	Moore DAJ (2006)	[11]	L-J PM	Peru	338/334	36	0	0	302	64	1	10	259	15
	Moore DAJ (2004)	[15]	MABA-MIC	Peru	276	31	7	2	236	63	8	10	195	ND
	Caviedes L (2000)	[16]	MABA-MIC	Peru	88	16	9	0	63	22	1	0	65	ND
	
	**TOTAL**				**1187/1182**	**198**	**35**	**8**	**946**	**320**	**37**	**27**	**798**	

**Genotype**^®^**MTBDR**	Hillemann D (2007)	[18]	L-J PM	Germany	71	30	1	1	39	36	0	5	30	ND
	Somoskovi A (2006)	[19]	BACT 460	USA	130	25	3	0	102	50	0	38	47	ND
	Hillemann D (2006)	[20]	L-J PM	Germany	42	15	0	0	27	17	0	0	25	ND
	
	**TOTAL**				**243**	**70**	**4**	**1**	**168**	**103**	**0**	**43**	**102**	

**Genotype**^®^**MTBDR*plus***	Causse M (2008)	[21]	MGIT 960	Spain	18	9	0	0	9	8	0	0	10	ND
	Lacoma A (2008)	[22]	BACT 460	Spain	51	29	1	0	21	28	0	2	21	ND
	Miotto P (2008)	[23]	Sequencing	Italy	173/172	20	0	0	153	117	0	0	55	ND
	Barnard M (2008)	[24]	MGIT 960	S. Afrrica	454/452	94	2	1	357	114	1	7	330	2
	Hillemann D (2007)	[18]	L-J PM	Germany	71	30	1	1	39	37	0	4	30	ND
	
	**TOTAL**				**767/764**	**182**	**4**	**2**	**579**	**304**	**1**	**13**	**446**	

***Abbreviations: ***BACT 460 = Radiometric BACTEC 460, FS = false susceptible, FR – false resistant, L-J PM = Proportion method on Lowenstein-Jensen medium, MGIT = Mycobacterium growth indicator tube, MODS = Microscopic Observation drug susceptibility assay; ND = No data in study report, NRA = Nitrate Reductase Assay, Ref = Bibliographic reference, Time = Duration in days from setting the test to obtaining 100% of the results in the specified study, TS = true susceptible, TR = true resistant. The MODS assay has been studied on both smear positive and smear negative sputum samples with good results, which is not the case with any other tests.

### Quality of study reports (QUADAS analysis results)

Thirteen (72%) of the 18 study reports had reported the spectrum of patients or samples to be representative of those to benefit from routine use of the test (QUADAS item 1). Eight (44%) of the 18 study reports clearly described the patient or sample selection criteria (QUADAS item 2). Quality items 3 to 9 that relate to internal validity of the assay results were reported in 67–100% of the studies. Lastly, seven of the 18 studies reported on blinding to the results of the reference test (items 10) while five reported on blinding to the new tests results (item 11). Un-interpretable results were reported in 13 (78%) of the 18 studies (item 13).

### Sensitivity and specificity

#### Rifampicin

**Figure 1:**
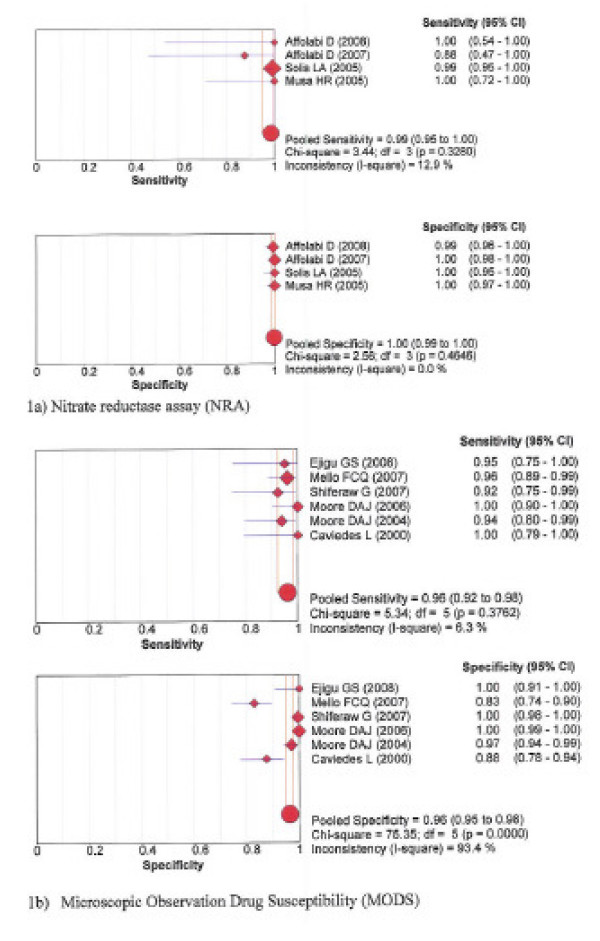
**Forest plots of sensitivity and specificity – Rifampicin phenotypic assays: 1a) Nitrate reductase assay; 1b) Microscopic Observation drug susceptibility**.

**Figure 2:**
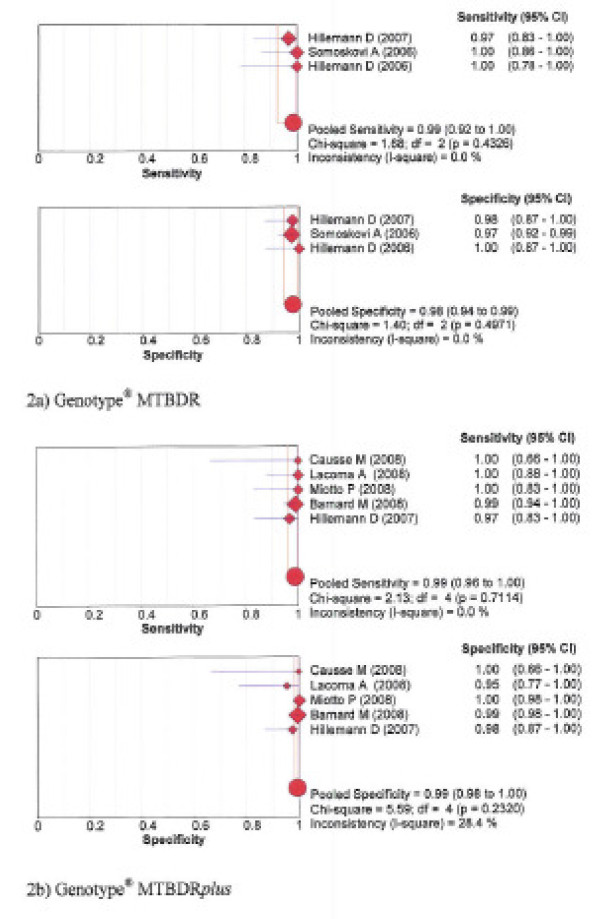
**Forest plots of sensitivity and specificity – Rifampicin Genotypic assays: 2a) Genotype^® ^MTBDR; 2b) Genotype^® ^MTBDR*plus***.

The pooled sensitivity and specificity for detection of resistance to rifampicin was 99% and 100% with NRA, 96% and 96% with MODS, 99% and 98% with Genotype^® ^MTBDR, and 99% and 99% with the new Genotype^® ^MTBDR*plus*, respectively. See forest plots figures 1 and 2.

#### Isoniazid

**Figure 3:**
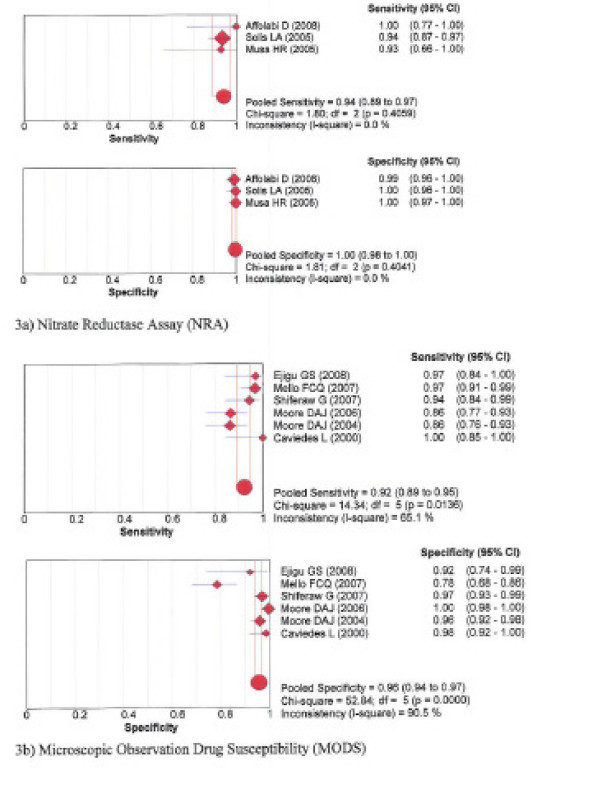
**Forest plots of sensitivity and specificity – Isoniazid phenotypic assays: 3a) Nitrate reductase assay; 3b) Microscopic Observation drug susceptibility**.

**Figure 4:**
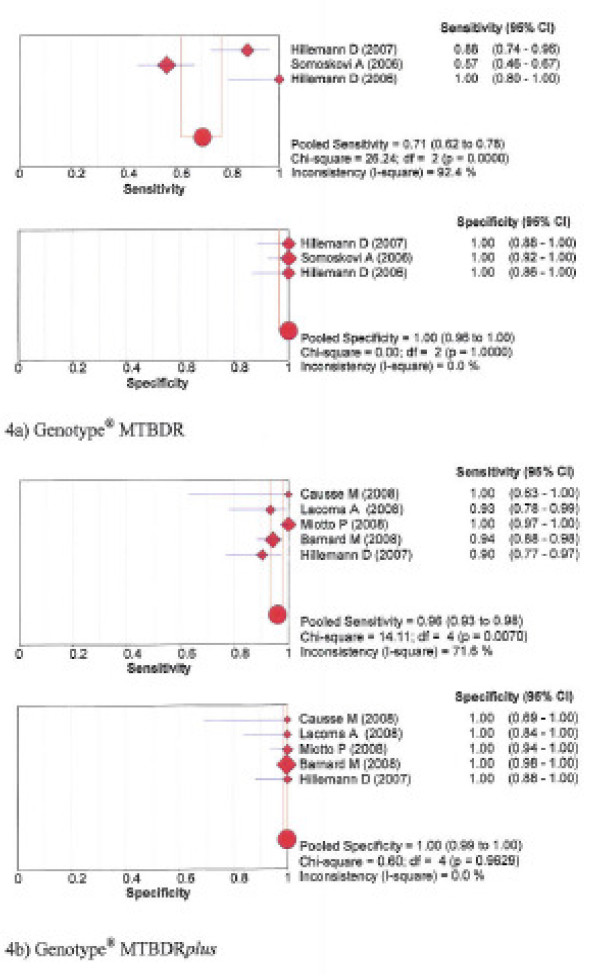
**Forest plots of sensitivity and specificity – Isoniazid Genotypic assays: 4a) Genotype^® ^MTBDR; 4b) Genotype^® ^MTBDR*plus***.

The pooled sensitivity and specificity for detection of resistance to isoniazid was 94% and 100% for NRA, 92% and 96% for MODS, 71% and 100% for Genotype^® ^MTBDR, and 96% and 100% with the Genotype^® ^MTBDR*plus*, respectively. See forest plots figures 3 and 4.

### Area under the sROC curve

**Figure 5:**
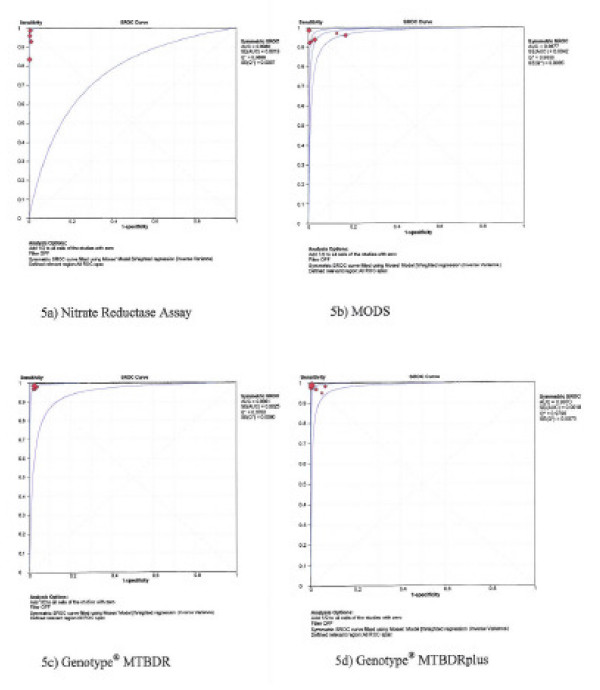
**Summary receiver operating characteristic (sROC) curves – rifampicin testing**.

**Figure 6:**
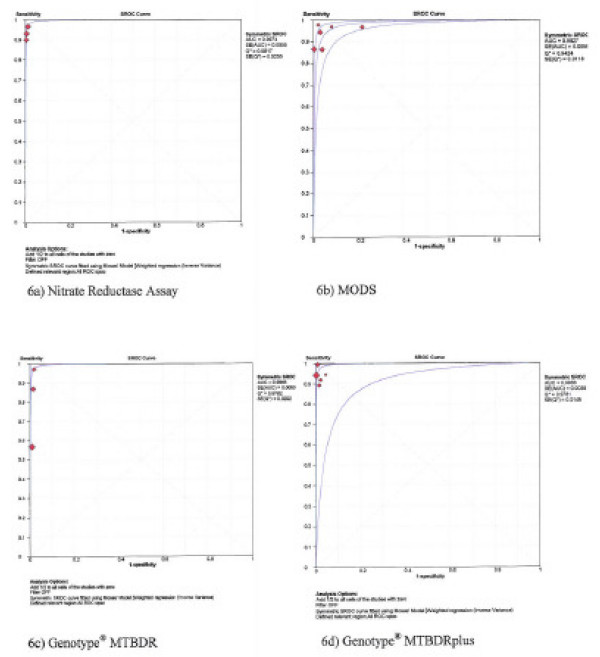
**Summary receiver operating characteristic (sROC) curves – isoniazid testing**.

The sROC curves are shown in figures 5 and 6 for rifampicin and isoniazid, respectively. The area under the sROC curves was 0.98 to 1.00 for each of the four tests for both rifampicin and isoniazid, and the Cochrane (Q*) index ranged from 0.95 to 0.99.

### Heterogeneity in sensitivity and specificity among study reports

Table 3 presents for each test the Spearman correlation coefficient between the logit of sensitivity and logit of 1-specificity. The degree of deviation of sensitivity and specificity estimates from the vertical line corresponding with the pooled estimates, the Chi-square *p*-values and inconsistence index (1-squared) are shown in the forest plots for each test, figures 1, 2, 3, 4.

**Table 3:** Spearman correlation coefficient Logit (sensitivity) vs Logit (1- specificity)

Test	Rifampicin		Isoniazid	
	
	Spearman correlation coefficient	p-value	Spearman correlation coefficient	p-value
Nitrate Reductase Assay	0.400	0.600	1.000	0.000
MODS	0.086	0.872	0.143	0.787
Genotype^® ^MTBDR	0.500	0.667	1.000	0.000
Genotype^® ^MTBDRplus	-0.200	0.747	-0.100	0.873

MODS = Microscopic Observation Drug Susceptibility. **Note: **A strong positive Spearman correlation coefficient between the logit of sensitivity and logit of 1-specificity suggests threshold/cutoff effect [27]. Since pooling sensitivity and specificity is more reliable in the absence of a threshold effect the NRA and the Genotype^® ^MTBDR assays should be primarily judged based on their areas under sROC, while the other tests can be reliably judged based on their pooled values.

### Time to Results (TTR)

The average time to 100% of the results was 23 days (range 18–28 days) for the NRA and 21 days (range 15–29) for MODS. One of the Genotype^® ^MTBDR*plus *studies reported TTR (2 days).

## Discussion

This study aimed at assessing the sensitivity, specificity, and time to results of the NRA, MODS, Genotype^® ^MTBDR and Genotype^® ^MTBDR*plus *tests for direct detection of resistance to rifampicin and isoniazid compared with conventional indirect DST. The results are intended to guide TB control programmes in RLS to select for further operational study highly sensitive and specific tests with shorter time to results for detection of MDR TB.

### Sensitivity and specificity of in-house phenotypic assays

Direct NRA performed with excellent pooled sensitivity and specificity for both rifampicin and isoniazid (94% – 100%). These findings indicate improved performance of the test when compared to results in a review by Martin A et al where sensitivity and specificity of direct NRA studies was 88% – 100% [28]. The MODS test with the highest number of direct DST studies showed good pooled sensitivity and specificity (86–100%) but performance of the MODS was slightly lower compared to the NRA.

### Sensitivity and specificity of the commercially available genotypic assays

Both the Genotype^® ^MTBDR and Genotype^® ^MTBDR*plus *showed excellent pooled sensitivity and specificity for detection of resistance to rifampicin (96–100%). For isoniazid, sensitivity of the Genotype^® ^MTBDR*plus *was high (96%; 95% CI 93–98%) but it was low with the Genotype^® ^MTBDR test (71%; 95% CI 62–78%). The pooled specificity was excellent for both assays *i.e*. 100%. The Genotype^® ^MTBDR test was designed to detect the most common mutations for INH resistance in the *katG *gene, and these account for 50–80% of INH resistance in *M. tuberculosis *[17]. The newer Genotype^® ^MTBDR*plus *detects additional mutations in the *katG *gene and also in the *inhA *promoter region for isoniazid resistance [18], leading to a higher sensitivity. Ling et al also found pooled sensitivity and specificity of 98% and 99%, respectively for rifampicin but for isoniazid sensitivity was 84%, though specificity was also 100% [29]. In their analysis, direct and indirect testing with both the old Genotype^® ^MTBDR and The Genotype^® ^MTBDR*plus *test was combined, and this could explain the higher sensitivity for detection of resistance to isoniazid in their study. In our study the pooled sensitivity for isoniazid resistance detection of 96% with the Genotype^® ^MTBDR*plus *alone means that this test performs excellent as a direct assay for INH as well. This is an advantage over the old Genotype^® ^MTBDR test, and the related test – the Line Probe Assay (INNO-LiPA Rif TB Assay; Innogenetics, Ghent, Belgium), which detects mutations in only the *rpoB *gene for rifampicin but not isoniazid resistance [23,24,30]

### Area under the sROC curve

Estimating mean sensitivity and specificity alone without looking at the area under the sROC curve may result into underestimation of test accuracy [31]. The area under the sROC, which is also an estimate of test accuracy was almost one for each test (see figures 5 and 6), meaning that the probability of the test to correctly rank a random pair of resistant and susceptible TB would be 98–100%. The Cochrane (Q*) index, *i.e*. the point at which the sROC crosses the diagonal line from the left upper coordinate to the right bottom coordinate was excellent for each of the four tests (see figures 5 and 6). At the Q* point, sensitivity is equal to specificity and the false positive rate is at the minimum [31]. With the high sensitivities, specificities, area under the sROC and Q* index, the diagnostic accuracy for the direct NRA, MODS, and Genotype^® ^MTBDR*plus *assays is high.

### Heterogeneity in accuracy estimates

Variations caused by threshold/cut off effect are detected by a Spearman correlation coefficient between the logit of sensitivity and logit of 1-specificity with a significant p-value [27,32,33]. The Spearman correlation coefficient was not significant in most of the tests (table 3) except for the NRA and the Genotype^® ^MTBDR assays for INH. To test for causes of variations other than threshold, visual inspection of the forest plots showed very minimal deviation of individual study estimates of sensitivity and specificity from the pooled value, except for the Genotype^® ^MTBDR for INH. Furthermore, the MODS and the two genotypic assays showed significant p-values for the Chi-square, and a high inconsistency index for INH (see forest plots). This implies that heterogeneity in sensitivity and specificity due to chance, study design/population, and the way a study was conducted could have caused the variations in the latter tests [34,35]. Since pooling sensitivity and specificity is more reliable in the absence of a threshold effect, the NRA and the Genotype^® ^MTBDR assays should be primarily judged based on their areas under sROC, while the other tests can be reliably judged based on their pooled values.

### Time to results (TTR)

We presented data for TTR for 100% of the DST results to permit comparison of rapidity between the different tests. For the MODS and NRA tests, the average TTR was within 23 days compared with the 2 months required for conventional indirect testing. Moreover, most results were ready in 7–14 days for MODS (data not shown). Contamination and indeterminate results in phenotypic methods may prolong the time to the final result but this was difficult to quantify in this study. For the genotypic assays, the only study that indicated TTR reported 2 days, but the protocol of these genotypic assays allows DST results within 1–2 days [36]. The risk of amplicon contamination is a problem in PCR-based tests. This could prolong the time to results as repeat testing or new samples have to be analysed. From this study however, it was evident that direct testing with any of the studied tests significantly shortens the time to detection of MDR TB, and would permit timely decision on patient management. This is supported by a retrospective study of the impact of direct MODS assay in a clinical setting where DST results in 82.8% of the cases were available before those of any standard method. In 41% of these cases, the rapid results should have prompted a timely modification in patient management [37].

Even though, the potential for contamination, resulting in un-interpretable or indeterminate results in phenotypic direct tests were to be considered, the time periods shown in this study were for 100% of the results. Additionally, traditional reservations about the direct versus indirect testing pertain in part to the inability to control the inoculum of a direct test. For rifampicin and isoniazid – the two important drugs defining MDR TB, it appears that inoculum size in direct assays is not as critical as previously believed. Moreover if MDR TB is identified then further DST, including second line drugs can be undertaken. It should be understood that the markedly shortened time to results with rapid direct testing is meaningless in settings where lengthy turn around time (TAT) is due to delays in sample delivery to the laboratory or delays in reporting the laboratory results. With that aside, it appears that the choice of which direct test to customize in a given setting will likely depend on some other operational issues such as technical ease, cost and bio safety that are briefly discussed below:

### Technical ease

The NRA and MODS are technically simple to perform and do not require sophiscated equipment when compared with the conventional proportion method on Lowenstein-Jensen (L-J) medium. The relative complexity of the PCR-based genotypic tests compared with the NRA and MODS may be a limitation to their use in resource-limited settings (RLS). Genotypic assays require well trained manpower though this is not as critical as previously believed [38]. Also required are equipment such as sonicators, thermocyclers, hybridization instruments, and a suitable laboratory infrastructure with unidirectional work flow to minimize contamination. These resources are not readily available in RLS and this could be a reason why none of the analysed genotypic studies was conducted in a typical RLS as shown in table 2.

### Cost per test

Due to insufficient data, planned cost analysis was not performed in this study. One report indicated MODS to cost $3 per sample while another report from S. Africa suggested that direct Genotype^® ^MTBDR*plus *assay would be 50% cheaper than conventional testing [12,24]. Direct testing with the MODS or the NRA is probably cheaper than molecular tests but since the time to results is shortest with genotypic assays a cost-effective analysis is warranted.

### Bio safety

Conventional indirect testing requires sophisticated bio safety level 3 laboratories with negative pressure air flow to safely manipulate grown cultures at the time of the DST. Conversely, direct DST is less demanding and the bio safety risk is similar to that for workers doing microscopy [39]. Direct DST may safely be performed in a TB lab with N-95 masks for personal protection and a biological safety cabinet as well as a laboratory door with a locker to stop airflow turbulence [12].

### Quality of analysed reports

The quality of the analysed reports was good in some but not all aspects according to QUADAS analysis [26]. Three-quarters of the study reports indicated the patient spectrum from whom the studied samples were obtained (quality item 1), which is essential for the applicability and specificity of the results. However, few studies indicated the selection/sampling techniques, and those which did used mostly convenience or biased sampling, casting uncertainty about overall representativity and applicability of the results. Prospective consecutive sampling of patients to whom the test will be used is recommended for diagnostic accuracy studies seeking to recommend any new test for routine use [40]. Among all the reports, two demonstrated the recommended design and conduct of a diagnostic study, and followed the standards for reporting diagnostic accuracy studies (STARD) [11,24,41]. Quality items 3–9 which relate to the internal validity of the results were excellent across all the analysed reports. It was unclear if blinding was done or not in most studies (items 10 and 11), but un-interpretable results (item 13), a key issue in direct testing were reported in almost eighty percent of the studies. Quality items 12 and 14 were excluded from analysis because the studies did not have a patient follow up component.

Combining high test performance and the operational issues discussed above, direct NRA and MODS assays appear to be competing tests for TB laboratories at safety level 2 in RLS. However, it is possible that in most of such settings, laboratories are familiar with the L-J solid medium-based assays, where NRA would require only a minor adjustment to be implemented in the routines.

Other tests that have recently appeared in the literature and proposed for TB high burden RLS include the Alamar blue, MTT (3-(4,5-dimethylthiazol-2-yl)-2,5-diphenyltetrazolium bromide), and resazurin assays [42]. Most of these studies were performed as indirect assays. For MTT and the manual mycobacterium growth indicator tube (MGIT; Becton Dickinson, Sparks, Maryland, we came across only two direct studies under each test [43-46]. These tests were excluded from the meta-analysis because the very few study reports made it difficult to give conclusive comments on the methods.

## Conclusion

Direct testing with the NRA, MODS and Genotype^® ^MTBDR*plus *for MDR TB is highly sensitive and specific, and significantly more rapid than conventional indirect susceptibility testing. The choice of which test to adopt will likely depend on technical ease and cost-effectiveness studies in the local settings, but the NRA and MODS appear to be promising tests for RLS.

### Study Limitations

Few direct DST reports were available for our analysis. This could be a limitation to generalization of the findings in this study. Second, since not all the reviewed studies fulfilled the study quality items in the QUADAS tool, the results in some of the analysed reports could have affected the pooled estimates shown in this study. However, some authors simply don't report according to standards for reporting diagnostic accuracy studies (STARD) even when the studies themselves were performed well.

## Competing interests

The authors declare that they have no competing interests.

## Authors' contributions

All the authors planned and designed the study. FB:Retrieved and reviewed the study reports, summarized and analysed the data, and prepared the manuscript. MH:Retrieved some of the study reports and critically revised the manuscript versions. SH:Critically revised the manuscript versions. MJ:Critically revised the manuscript versions.

## Pre-publication history

The pre-publication history for this paper can be accessed here:

http://www.biomedcentral.com/1471-2334/9/67/prepub
